# The effects of early high-volume hemofiltration on prolonged cardiac arrest in rats with reperfusion by cardiopulmonary bypass: a randomized controlled animal study

**DOI:** 10.1186/s40635-016-0101-6

**Published:** 2016-09-09

**Authors:** Koichiro Shinozaki, Joshua W. Lampe, Junhwan Kim, Tai Yin, Tong Da, Shigeto Oda, Hiroyuki Hirasawa, Lance B. Becker

**Affiliations:** 1 The Feinstein Institute for Medical Research, Northwell Health System, 350 Community Dr., Manhasset, NY 11030 USA; 2 Department of Emergency and Critical Care Medicine, Chiba University, Chiba, Japan; 3 Center for Cellular Immunotherapies, the University of Pennsylvania, Philadelphia, PA USA

**Keywords:** Renal replacement therapy, High-volume hemofiltration, Cardiac arrest, Cytokine, Interleukin 6, Cardiopulmonary bypass

## Abstract

**Background:**

It is not yet clear whether hemofiltration can reduce blood cytokine levels sufficiently to benefit patients who suffer prolonged cardiac arrest (CA) treated with cardiopulmonary bypass (CPB). We sought to assess effects of high-volume and standard volume continuous veno-venous hemofiltration (CVVH) on blood cytokine levels and survival in a rat model of prolonged CA treated with CPB.

**Methods:**

Sprague-Dawley male rats were subjected to 12 min of asphyxia to induce CA. CPB was initiated for resuscitation of animals and maintained for 30 min. Twenty-four rats were randomly assigned into three groups: without CVVH treatment (sham); standard volume CVVH at a filtration rate of 35–45 mL/kg/h; and high-volume hemofiltration (HVHF, 105–135 mL/kg/h). Hemofiltration was started simultaneously with CPB and maintained for 6 h. Plasma TNFα and IL-6 levels were measured at baseline, 0.5, 1, 2, 3, and 6 h after reperfusion. Survival time, neurological deficit score, and hemodynamic status were assessed.

**Results:**

All animals survived over 6 h and died within 24 h. There were no significant differences in survival time (log-rank test, sham vs. CVVH; *p* = 0.49, sham vs. HVHF; *p* = 0.33) or neurological deficit scores (ANOVA, *p* = 0.14) between the groups. There were no significant differences in blood cytokine levels between the groups. Mean blood pressure in sham group animals increased to 1.5-fold higher than baseline levels at 30 min. HVHF significantly reduced blood pressure to 0.7-fold of sham group (*p* < 0.01).

**Conclusions:**

There was no improvement in mortality, neurological dysfunction, TNFα, or IL-6 levels in rats after prolonged CA with CPB on either hemofiltration group when compared to the sham group.

**Electronic supplementary material:**

The online version of this article (doi:10.1186/s40635-016-0101-6) contains supplementary material, which is available to authorized users.

## Background

Cardiopulmonary bypass (CPB) has been shown to rescue victims from refractory cardiac arrest (CA) [1, 2], in which a patient suffers a prolonged time of whole-body ischemia. CPB produces more blood flow than conventional cardiopulmonary resuscitation (CPR) allowing the return of spontaneous circulation (ROSC) even after a prolonged period of CA [2, 3]. CPB is, therefore, considered indispensable for refractory CA patients [3].

Whole-body ischemia/reperfusion injury is thought to activate the inflammatory response, which increases the risk of death in patients after CA [4, 5]. Therefore, a method to regulate the highly activated systematic inflammatory response that occurs after refractory CA with CPB is of growing interest in post-cardiac arrest syndrome (PCAS) [4, 6–8].

Inflammatory cytokines, such as TNFα and interleukin-6 (IL-6), are key mediators not only of sepsis [9–11] but also of PCAS [4]. Several extracorporeal filtering techniques have been used to remove these cytokines [12, 13]. Continuous veno-venous hemofiltration (CVVH) is one of the emerging techniques that has been studied for this purpose [14–17]. Therefore, CVVH is a possible adjunct therapy for refractory CA treated with CPB [2]. However, it is not yet clear whether CVVH is effective in treating patients with hypercytokinemia who undergo CPB after refractory CA. Therefore, the clinical application of CVVH to critically injured patients with ischemia/reperfusion is still controversial [18, 19].

There have been relatively few reports that study CVVH on CA with CPB. Nagashima et al. [20] reported the effect of super high-volume hemofiltration (filtration rates; 300 mL/kg/h) on the improvement of vascular permeability and the regulation of the inflammatory response in the lung using experimental animals with CA and CPB. However, hemofiltration volumes greater than 200 mL/kg/h are logistically difficult to perform in clinical settings, because it requires special equipment to supply the large amount of replacement fluid [21]. Combes et al. [18] recently reported that early high-volume hemofiltration (filtration rates: 80 mL/kg/h) did not impact any clinical outcomes of the patients who were in shock after cardiac surgery with CPB. These conflicting results on clinical benefits raise an important question whether hemofiltration at a clinically acceptable range of filtration volume (less than 120 mL/kg/h) effectively reduces the blood levels of cytokines in refractory CA patients treated with CPB.

Therefore, we tested whether it is beneficial to use CVVH at high (105–135 mL/kg/h) or standard (35–45 mL/kg/h) hemofiltration volumes in a rodent model with prolonged CA and CPB. Furthermore, we investigated the effect of two differing hemofiltration settings on blood TNFα and IL-6 levels after CA.

## Methods

The study protocol was approved by the Institutional Animal Care and Use Committee of the University of Pennsylvania. All the instrumentation was performed according to the previously described protocol [22–24]. In brief, 24 adult male Sprague-Dawley rats (450–550 g, Charles River Laboratories, Wilmington, MA) were anesthetized with 4 % isoflurane (Isosthesia, Butler-Schein AHS, Dublin, OH, USA) and intubated with a 14-gauge plastic catheter (Surflo, Terumo Medical Corporation, Somerset, NJ, USA). Anesthesia was maintained with 30 % oxygen and 2 % isoflurane to sustain a surgical depth of anesthesia. Animals were mechanically ventilated (Ventilator Model 683, Harvard Apparatus, Holliston, MA, USA) at a tidal volume (TV) of 8 mL/kg and a respiratory rate of 40 to 45 breaths/min, which was adjusted to maintain an EtCO_2_ between 35 and 45 mmHg (Micro-Capnometer, Columbus Instruments, Columbus, OH, USA). One hundred percent oxygen was supplied to the animals during CPB. We returned the oxygen supply to baseline levels (30 %) 90 min after CPB.

The core temperature was maintained at 37° centigrade +/−0.5° using an esophageal temperature probe for continuous temperature monitoring. During the CPB session, the temperature was controlled using the CPB warmer circuit attached to an oxygenator. For the period of surgical preparation and the CVVH operation, a warming blanket and a heating lamp were used to control the temperature as the CPB circuit was not available. The left femoral artery was cannulated (sterile polyethylene-50 catheter inserted for 2 cm) for continuous arterial pressure monitoring (MLT844, ADInstruments; Bridge Amplifier ML221, ADInstruments, Colorado Springs, CO, USA). The left femoral vein was cannulated with a 20-gauge catheter cannula (Insyte-W, BD, Franklin Lakes, NJ, USA) for the blood inflow of CVVH. The right femoral artery was cannulated with a 20-gauge catheter cannula for the blood inflow of CPB. The right internal jugular vein was cannulated for the venous outflow of CPB and CVVH with a modified 4-hole 14-gauge catheter (Surflo, Terumo Medical Corporation, Somerset, NJ, USA), which was advanced into the vena cava. After the animals were disconnected from the mechanical ventilator, buprenorphine was administered via subcutaneous injection of 0.05 mg/kg buprenex (Buprenex, Reckitt Benckiser Pharmaceuticals, Inc., VA, USA).

### Cardiac arrest and reperfusion using cardiopulmonary bypass

After instrumentation, neuromuscular blockade was achieved by slow intravenous administration of 2 mg/kg of vecuronium bromide (Vecuronium Bromide, Hospira, Lake Forest, IL, USA). CA was induced via asphyxiation by switching off the ventilator. CA was defined as a mean arterial pressure (MAP) of less than 20 mmHg. After 12 min of asphyxia, mechanical ventilation was restarted and CPB was started. Activated coagulation time (ACT) was kept above 250 s by administering heparin (Heparin, SAGENT Pharmaceuticals, Schaumburg, IL, USA). The CPB circuit was primed using a total volume of 21.1 mL priming solution composed of 10 mL of Plasma-Lyte A (Baxter, Deerfield, IL, USA), 10 mL of 6 % Hetastarch (Hospira, Lake Forest, IL, USA), 0.8 mL of 0.406 mEq/mL magnesium sulfate (Magnesium Sulfate, APP Pharmaceuticals, Schaumburg, IL, USA), and 0.3 mL of 3.3 mmol/mL THAM Solution (XVIVO Perfusion AB, Göteborg, Sweden). The hemofiltration circuit was primed with the same solution using a priming volume of 6.9 mL. CPB was manipulated for a total of 30 min in all animals. Vasopressors or inotropic agents were not injected. At the end of CPB operation, the remaining blood in the circuit was collected and slowly reinfused into the animal.

### Experimental protocol and continuous veno-venous hemofiltration settings

Animals were randomized into three groups: (a) treated with a standard filtration rate of CVVH (*n* = 8); (b) treated with a high filtration rate of HVHF (*n* = 8); and (c) sham (without CVVH or HVHF but with surgery, CA, and CPB; *n* = 8). CVVH was started simultaneously with CPB (Additional file 1: Figure E1). The blood flow for veno-venous hemofiltration was controlled at a rate of 3 mL/min (5.5 to 6.5 mL/min/kg) (Additional file 2: Figure E2a and b) in animals treated with either CVVH or HVHF. At the end of 30-min CPB, the CPB circuit was removed and hemofiltration was restarted at a blood flow rate of 3 mL/min (5.5 to 6.5 mL/min/kg), which was maintained for 5.5 h. The filtration rates were controlled at two settings, 20 mL/h (35 to 45 mL/h/kg) in the CVVH group and 60 mL/h (105 to 135 mL/h/kg) in the HVHF group. The effluent was drawn from a side hole of the hemofilter using a drawing pump (Genie Touch Syringe Pump, Kent Scientific, Torrington, CT, USA). Replacement fluid, added at the same rate of effluent, was infused through the catheter inserted into the left femoral vein (Additional file 2: Figure 2a and b, post-dilution, zero-balanced filtration). The investigated hollow-fiber hemofilter was AN69ST with an in vivo cut-off value of 35–40 kD (GAMBRO Industries, Meyzieu, France). PrismaSATE BGK 4/2.5 (Gambro Renal Products, Daytona Beach, FL, USA) was used as the replacement fluid. All surviving rats were extubated at 7 h of operation (Additional file 1: Figure E1), and then, animals were observed for another 5 h.

### Measurements and calculations

The neurologic deficit score (NDS, 0–100 %; 0 %, normal; 100 %, brain-dead) that has been described previously [23–25] was assessed in all rats after extubation before buprenorphine was injected. The assessment was performed by the investigators, and it was not blinded from the procedures. Blood samples of 0.5 mL were obtained from the left femoral catheter at baseline before CA, at 30 min, 1, 2, 3, and 6 h after resuscitation. Effluent samples of 1.0 mL were obtained at 1, 2, 3, and 6 h after resuscitation, which contained the cumulative amount of the effluent samples during 0–1, 1–2, 2–3, and 3–6 h. MAP was monitored continuously through 6 h after resuscitation. Blood gas analysis (pH, pO_2_, pCO_2_; i-STAT, Heska, East Windsor, NJ, USA), (Additional file 3: Figure E4), laboratory data (ACT, sodium, potassium, blood urea nitrogen (BUN), glucose, lactate; i-STAT, Heska, East Windsor, NJ, USA), and a hematocrit (Stat Spin MP, Iris Sample Processing, Westwood, MA, USA) were measured immediately after blood sampling. The remaining blood was centrifuged at 1000 g for 10 min, and the isolated plasma was stored at −80 °C until thawed for cytokine assays. Concentrations of TNFα and IL-6 in plasma and effluent samples were measured using an enzyme-linked immunosorbent assay kit specific for rats according to manufacture instructions (R&D Systems, Inc. Minneapolis, MN, USA). The density was determined using a microplate multimode reader (Turner Biosystems, Sunnyvale, CA, USA) set at 450 nm. The cumulative amount of removed cytokines was calculated from the effluent concentration of both cytokines multiplied by filtrated volume.

### Statistical analysis

Continuous data that were normally distributed were reported as mean and standard deviation. Data that were not normally distributed were shown as median and inter-quartile range. Group comparisons were first made using one-way analysis of variance followed by Scheffe’s test as a post hoc test. TNFα and IL-6 data were not normally distributed; therefore, we compared the data after logarithmic transformation. Mean +/− standard deviation of the logarithmic transformed data is reported in figures. The Kaplan-Meier method was used to establish the 12-h survival plot, and probability values for survival comparisons were calculated using the log-rank statistic. A two-tailed *p* value of <0.05 was considered statistically significant. All calculations were performed with SPSS Statistics Ver. 22 for Mac (IBM Corp., Armonk, NY).

## Results

In total, 24 animals were subjected to CA and resuscitated using CPB. Those animals were randomized into the sham (*n* = 8), the CVVH (*n* = 8), and the HVHF (*n* = 8) groups. Basal characteristics and resuscitation data are shown in Additional file 4: Table E1. There were no significant differences between groups. All animals were successfully resuscitated by CPB and survived more than 6 h after resuscitation. Therefore, hemofiltration was successfully performed for 6 h in animals that were assigned to the CVVH or HVHF group.

It is difficult to determine the cause of death in animals. Arterial pressure and ECG were recorded from 20 animals (83 %) until animals died. The data indicated that some animals had seizures in advance of the death whereas all animals had hemodynamic collapse before they died. Relations between the seizures and the hemodynamic collapse were not clear. No lethal arrhythmias of VF rhythms were found. Before and after hemofiltration, there was no evidence of surgical complications, such as major bleeding.

### Effect of continuous veno-venous hemofiltration on survival and neurological outcomes

**Fig. 1:**
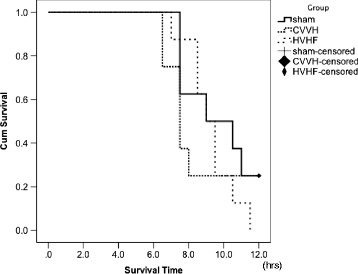
Short-term survival after resuscitation compared between the three experimental groups. Continuous veno-venous hemofiltration therapy did not improve survival in both settings of hemofiltration (analyzed by log rank; sham vs. CVVH, *p* = 0.49; sham vs. HVHF, *p* = 0.33). CVVH indicates standard volume continuous veno-venouse hemofiltration; *HVHF* high-volume hemofiltration

**Fig. 2:**
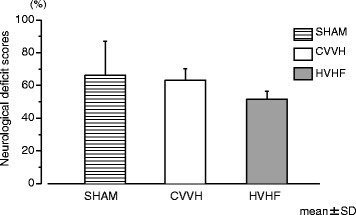
Neurological deficit scores at 7 h after resuscitation compared between the three experimental groups. Continuous veno-venous hemofiltration therapy did not improve neurological function at 7 h after resuscitation in both settings of hemofiltration (analyzed by ANOVA *p* = 0.135). Neurological deficit score is ranged between 0 and 100 %: 0 %, normal, and 100 %, brain-dead. CVVH indicates standard volume continuous veno-venouse hemofiltration; *HVHF* high-volume hemofiltration

Two animals in the CVVH group and one animal in the HVHF group died before extubation. All the other animals died after extubation. Figure 1 contains a survival curve for all animals. There were no significant differences in short-term survival outcomes between the experimental groups (log rank; sham vs. CVVH, *p* = 0.49; sham vs. HVHF, *p* = 0.33). Neurological deficit scores 7 h after resuscitation are displayed in Fig. 2. There were no significant differences between the groups (ANOVA; *p* = 0.135).

### Effect of continuous veno-venous hemofiltration on plasma cytokine levels

**Fig. 3:**
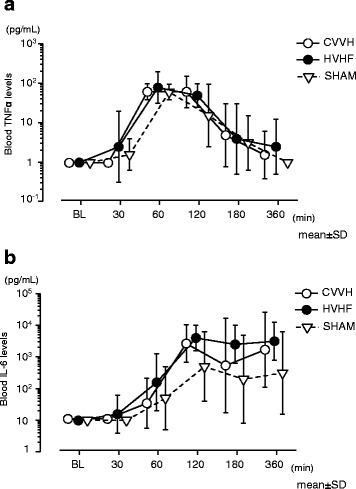
**a** Blood TNFα levels as a function of time compared between the three experimental groups. Continuous veno-venous hemofiltration therapy did not affect blood levels of TNFα during 6 h after resuscitation (analyzed by ANOVA *p* = 0.302, 0.709, 0.061, 0.804, and 0.351 at 30, 60, 120, 180, and 360 min after resuscitation, respectively). CVVH indicates standard volume continuous veno-venouse hemofiltration; *HVHF* high-volume hemofiltration. **b** Blood IL-6 levels as a function of time compared between the three experimental groups. Continuous veno-venous hemofiltration therapy did not affect blood levels of IL-6 during 6 h after resuscitation (analyzed by ANOVA *p* = 0.385, 0.444, 0.069, 0.188, and 0.228 at 30, 60, 120, 180, and 360 min after resuscitation, respectively). CVVH indicates standard volume continuous veno-venouse hemofiltration; *HVHF* high-volume hemofiltration

**Fig. 4:**
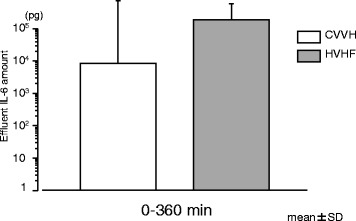
Cumulative amount of IL-6 in effluent removed from the circulating blood. CVVH indicates standard volume continuous veno-venouse hemofiltration; *HVHF* high-volume hemofiltration

Plasma TNFα and IL-6 levels were under detection limit in any baseline samples. Median plasma levels of TNFα increased to 60 (43–91) pg/mL (sham), 61 (52–88) pg/mL (CVVH), and 84 (48–130) pg/mL (HVHF) at 1 h, respectively, and then dropped to undetectable levels at 6 h. Median plasma levels of IL-6 increased to 967 (151–4336), 2308 (919–10,396), and 4765 (1725–10,557) pg/mL at 2 h, respectively, and high levels of IL-6 continued through 6 h. There were no significant differences in plasma levels of TNFα or IL-6 between three groups at any time (Fig. 3a, b). A cumulative amount of IL-6 in effluent samples obtained by two differing settings of CVVH is shown in Fig. 4. IL-6 was removed by both settings of CVVH. TNFα was not detected in most of the effluent samples (data not reported in detail).

### Effect of continuous veno-venous hemofiltration on arterial blood pressure

**Fig. 5:**
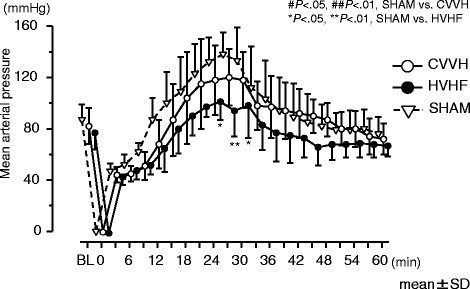
Mean arterial pressure during the first 1 h after resuscitation compared between the three experimental groups. MAP of three groups increased above baseline levels shortly after the return of spontaneous circulation. MAP of the HVHF group at 24, 27, and 30 min after resuscitation was significantly lower than the sham group (*p* < 0.05, *p* < 0.01, *p* < 0.05, respectively). CVVH indicates standard volume continuous veno-venouse hemofiltration; *HVHF* high-volume hemofiltration

MAP during the first hour of post-CA is shown in Fig. 5. MAP measurement takes at 30-min mark for all three groups. In the sham group, MAP elevated 1.53-fold higher than the baseline (133 ± 26 mmHg). In the second group, CVVH, MAP increased 1.44-fold higher than the baseline (118 ± 22 mmHg). In the third group, HVHF, MAP increased 1.27-fold higher than the baseline (98 ± 25 mmHg). The peak MAP during 1-h period of post-CA was significantly reduced in the HVHF group compared to that of the sham group (*p* < 0.01). These results indicate that HVHF sufficiently reduced increased MAP in early post-CA. There were no significant differences in MAP between the three groups following this time.

## Discussion

In order to understand the effect of hemofiltration rates on blood cytokine levels and outcomes of refractory CA victims, we evaluated the efficacy of high (105–135 mL/kg/h) or standard (35–45 mL/kg/h) filtration rates of CVVH on blood cytokine levels and survival in rats after prolonged CA treated with CPB. To our knowledge, this is the first study evaluating the effect of two filtration rates of CVVH using this model. In this study, we did not find significant differences in survival outcomes or peripheral blood levels of TNFα and IL-6 between the experimental three groups.

TNFα, one of the pivotal ligands in death receptor pathway of apoptosis [26], is an inflammatory cytokine that can induce IL-6 expression [27, 28]. It is thought that these cytokines are associated with outcomes in sepsis [28, 29]. The molecular weight of monomeric TNFα is 17 kD and that of IL-6 is 25 kD [30]. Our experimental hemofilter, with which internal diameter of the fiber is 240 μm, is reported to have an in vivo cutoff value of 35–40 kD [31]. CVVH using this filter is likely to contribute for removing TNFα and IL-6 from the circulating blood. Callaway et al. [7], using a rat model, found that 8 min of asphyxia triggered the release of inflammatory cytokines to the peripheral blood. It was also reported that therapeutic hypothermia did not alter the inflammatory response in cytokine levels. The possible explanation for the lack of effect of hypothermia was that 8 min of asphyxia was not long enough to demonstrate deleterious effects of systematic cytokine release. For this reason, 12 min of asphyxia time was applied in our study. As expected, a considerable amount of both cytokines, TNFα and IL-6, were detected in the circulating blood in our animal model.

However, we did not find any differences in blood levels of TNFα or IL-6 compared between the experimental three groups (Fig. 3). Data from our study raised two questions: (a) was CVVH able to remove these molecules from the blood? and (b) were the filtration rates sufficient enough to show its efficacy?

To determine if CVVH could remove cytokines from the blood, we investigated the sieving coefficient (SC) of the investigational hemofilter, AN69ST, on IL-6 (Additional file 4: Table E2). This result supports that CVVH operation can successfully remove IL-6 for a period of 6 h, keeping the SC to approximately 0.3.

To determine if the filtration rates were sufficient, we made a model for a prediction of blood IL-6 levels (Additional file 5: Figure E3). On the basis of this estimate, assuming no extra-IL-6 generation relating to the CVVH operation, blood IL-6 levels were expected to slightly decrease. In addition, because the investigational hemofilter of AN69ST is highly biocompatible, this filter is thought to have some adsorptive abilities to increase the cytokine removal [21, 30, 32].

**Fig. 6:**
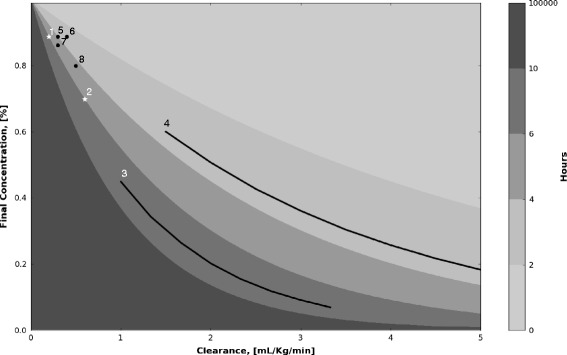
Final blood concentration of targeted molecules by clearance and operating time of hemofiltration. Assuming no generation of targeted molecules, the figure estimates the blood concentration at the end of hemofiltration expressed as a percentage of the initial concentration. These numbers are calculated by clearance (mL/kg/min) and operating time (h) of hemofiltration. The calculation was derived from the following general equation including clearance, operation time of hemofiltration, and the blood concentration of molecules [40]: $C={C}_0\cdot {e}^{-\frac{K\cdot t}{V}}$, where *C* is the blood concentration at the end of hemofiltration and *C*_0_ (at the beginning) is set at 1. *V* (mL/kg) is the total body water assumed that the body was 60 % water by weight (600 mL/kg), *t* is operational time (min) of hemofiltration, and *K* (mL/kg/min) is the clearance of investigated filters that can be replaced with SC (sieving coefficient) × Qf (filtration rates, mL/kg/min) [41] in the filtration technique. A period of time was given as a range of 0–2, 2–4, 4–6, 6–10, and 10–infinity hours in this figure (expressed as *gray color* gradation). Marks numbering from *1* to *8* displayed referenced reports on IL-6 removal; *1* and *2* are referring to two differing settings of our study (*1* is for standard volume hemofiltration and *2* is for high-volume hemofiltration); *3*, Laurent et al. [19] and *4*, Nagashima et al. [20], studies on whole-body ischemia/reperfusion injury; *5*, Kellum et al. [34], *6*, Bellomo et al. [21], *7*, Lu et al. [35], and *8*, Rogiers et al. [33], studies on a sepsis animal model. The studies, *1*, *2*, and *5*–*8*, used the same filter of AN69; therefore, SC was set at 0.3 according to our pilot data. For the other studies, *3* and *4*, SC was arbitrarily set at 0.3–1.0 because of the larger porosity of their investigated filters, which are made from high-flux hollow-fibers

Other researchers have previously examined the effect of CVVH using several hemofiltration settings in animal models or human clinical study [19–21, 33–35]. All of these studies succeeded in showing the beneficial effects of CVVH. Figure 6 shows clearance (K) × time of IL-6 removal for all these studies including our present work. According to the figure, the study on resuscitated CA patients [19] and ischemia animals followed by reperfusion with CPB [20] used a greater setting of Kt than our study. The difference in Kt values could explain the confliction of results in our study with others. Consistently, Combes et al. [18] recently reported no beneficial impacts of early high-volume hemofiltration at a relatively low filtration rate of 80 mL/kg/h on clinical outcomes of shock patients after cardiac surgery with CPB. The results might be explained by insufficient rates of filtration volume. Therefore, it could be inferred that a large Kt, greater filtration volume, and/or longer operation time might be needed to treat hypercytokinemia after global ischemia/reperfusion injury with CPB, which may benefit patient survival.

Kellum et al. [34] reported blood IL-6 levels during the hemofiltration in sepsis. They showed significantly lower blood IL-6 levels (17 pg/mL) in hemofiltration-treated animals than non-treated animals (62 pg/mL) using the cecal ligation and puncture model of rats. Blood IL-6 levels of their study are much lower than those of our study. Therefore, it appears that the reported sepsis models, where hemofiltration was effective, may have resulted in lower cytokine levels when compared to our injury model.

We found that increased MAP was significantly reduced in the HVHF group during the first 60 min after CPB. The finding, elevation of MAP with ischemia/reperfusion, was consistent with previous reports. Grootendorst et al. [36] reported that MAP increased during superior mesenteric artery clamping, and it was improved by hemofiltration. Also, Lu et al. [35] found that hemofiltration attenuated the increased plasma levels of dopamine and endothelin-1 in a septic shock animal model. Therefore, it is inferred in our study that HVHF might remove excess vaso-constrictive mediators from the circulating blood, and increased MAP was improved as a result. However, our study was not designed to study the hemodynamic effect of CVVH. Therefore, further studies will be necessary to validate an effectiveness of HVHF on humoral mediators of the cardiovascular system in whole-body ischemia/reperfusion injury.

There are several important limitations in our study. First, our model is different than the typical clinical case of CA treated with conventional CPR. In clinical situations, conventional CPR is immediately initiated when the patient has a CA. After a certain period of CPR, CPB might be implemented if required/possible [3]. However, to avoid possible confounders in animal models associated with CPR, such as inconsistent blood circulation during CPR, case-by-case pattern of ROSC achievement, and inconsistency of ROSC rates, we did not include CPR in our model. Although our model did not exactly mimic clinical practice, our model provides sufficient platform to test the potential therapeutic effects of CVVH on patients after refractory CA treated with CPB.

Secondly, CPB itself might activate inflammatory responses [37]. Therefore, our small rodent injury model might have greater inflammatory responses as compared to a typical CA plus CPR model in rodents [38, 39]. It is difficult to distinguish whether CA, CPB, or both are responsible for the inflammatory response after CA in our model. Therefore, this can be the potential reason that the treatment groups did not show benefits on outcomes. Future studies may wish to consider effects of CVVH or HVHF using a CA model with conventional CPR.

The sample size of this study is not adequate to detect the statistical significance of differences in survival outcomes between the groups. The outcome assessment was not blinded from the procedures, and it might include potential bias of this study. And also, technical difficulties of HVHF, such as increased friction fraction rates due to limited blood flow from small animals, might lessen the benefit of hemofiltration.

Lastly, as compared to clinical practice, we used a shorter period of CVVH operation and post-CA treatment, such as the intubation and mechanical ventilation support. Therefore, according to Kt/V theory, it might be possible that a longer period of CVVH could affect outcomes if the adequate time of post-CA treatment is combined. All animals developed anemia after CPB reperfusion. However, as we previously mentioned [23], the anemia was not an intentional part of the model in this trial. In addition, although a CVVH circuit potentially aggravated the anemia, we did not find any differences in hematocrit values between the groups at any time (data were not shown).

## Conclusions

Neither standard volume continuous veno-venous hemofiltration nor high-volume hemofiltration had any effect on blood TNFα or IL-6 levels in a rodent model of prolonged CA treated with CPB. In this study, we did not find beneficial effects of hemofiltration on neurological or survival outcomes of the animals.

## Additional files

Additional file 1: Figure E1. Experimental Protocol. Flow rates shown in this figure were typical of our experiments. ICU indicates intensive care unit; CPB, cardiopulmonary bypass; CVVH, continuous veno-venous hemofiltration. (PPTX 68 kb) Additional file 2: Figure E2a. Schematic of the rodent continuous veno-venous hemofiltration circuit combined with emergency cardiopulmonary bypass circuit. The letters indicate the location of monitoring and circuit components; A, out flow tube for two extra corporeal circuits; B, venous reservoir; C, roller pump; D, oxygenator of cardiopulmonary bypass; E, inflow tube for cardiopulmonary bypass; F, outflow for continuous venovenous hemofiltration; G, flow sensor; H, pressure sensor at pre-filter; I, pressure sensor at side hole; J, filtration (effluent) pump; K, screw clamp; L, inflow tube for continuous venovenous hemofiltration; M, replacement infusion pump. **Figure E2b**. Schematic of the rodent continuous veno-venous hemofiltration circuit. The letters indicate the location of monitoring and circuit components; A, roller pump; B, outflow for continuous venovenous hemofiltration; C, pressure sensor at pre-filter; D, pressure sensor at side hole; E, filtration (effluent) pump; F, screw clamp; G, inflow tube for continuous venovenous hemofiltration; H, replacement infusion pump.Flow rates demonstrated in schematic were the typical settings of two extra corporeal circuits. Filtration and replacement fluid rates in the CVVH group were 20 mL/h and those in the HVHF group were 60 mL/h. (PPTX 563 kb) Additional file 3: Figure E4 a and b. Blood gas analysis (PaO_2_ and PaCO_2_) as a function of time compared between the three experimental groups. There were no differences between the three groups. (PPTX 131 kb) Additional file 4: Additional manuscript. The contents include methods and results of "Estimated Blood Levels of IL-6 during CVVH Operation", "Blood Gas Analysis", "**Table E1**. Basal characteristics and resuscitation data of randomized animals" and "**Table E2**. Efficacy of hemofilter on IL-6 removal from circulating blood". (DOCX 305 kb) Additional file 5: Figure E3. Predicted blood levels of IL-6 compared between differing settings of hemofiltration. This estimate was based on mean blood levels of sham animals in this study. The sigmoid trend curve was generated by fitting the two-parameter logistic curve to our data. Sieving coefficient of our investigated hemofilter was set at 0.3. We assumed that a total volume of 600 mL was purified using CVVH in our model. This number was made by a calculation of 1000 g × 60 %, where 1000 g was an unit weight (kg) and 60 % was a rate of body water contained in the animal body. (PPTX 93.4 kb)

## Abbreviations

ACT: Activated coagulation time
CA: Cardiac arrest
CPB: Cardiopulmonary bypass
CPR: Cardiopulmonary resuscitation
CVVH: Continuous veno-venouse hemofiltration
HVHF: High-volume hemofiltration
IL-6: Interleukin-6
MAP: Mean arterial pressure
PCAS: Post-cardiac arrest syndrome
ROSC: Return of spontaneous circulation
SC: Sieving coefficient
