# Supplementary material for: The effects of early high-volume hemofiltration on prolonged cardiac arrest in rats with reperfusion by cardiopulmonary bypass: a randomized controlled animal study
Source: Intensive Care Med Exp. 2016 Sep 9;4(1):25. doi: 10.1186/s40635-016-0101-6 (PMC5017966; doi:10.1186/s40635-016-0101-6)
Supplement: Additional file 1: Figure E1. — Experimental Protocol. Flow rates shown in this figure were typical of our experiments. ICU indicates intensive care unit; CPB, cardiopulmonary bypass; CVVH, continuous veno-venous hemofiltration. (PPTX 68 kb) [file 40635_2016_101_MOESM1_ESM.pptx]

## Slide 1
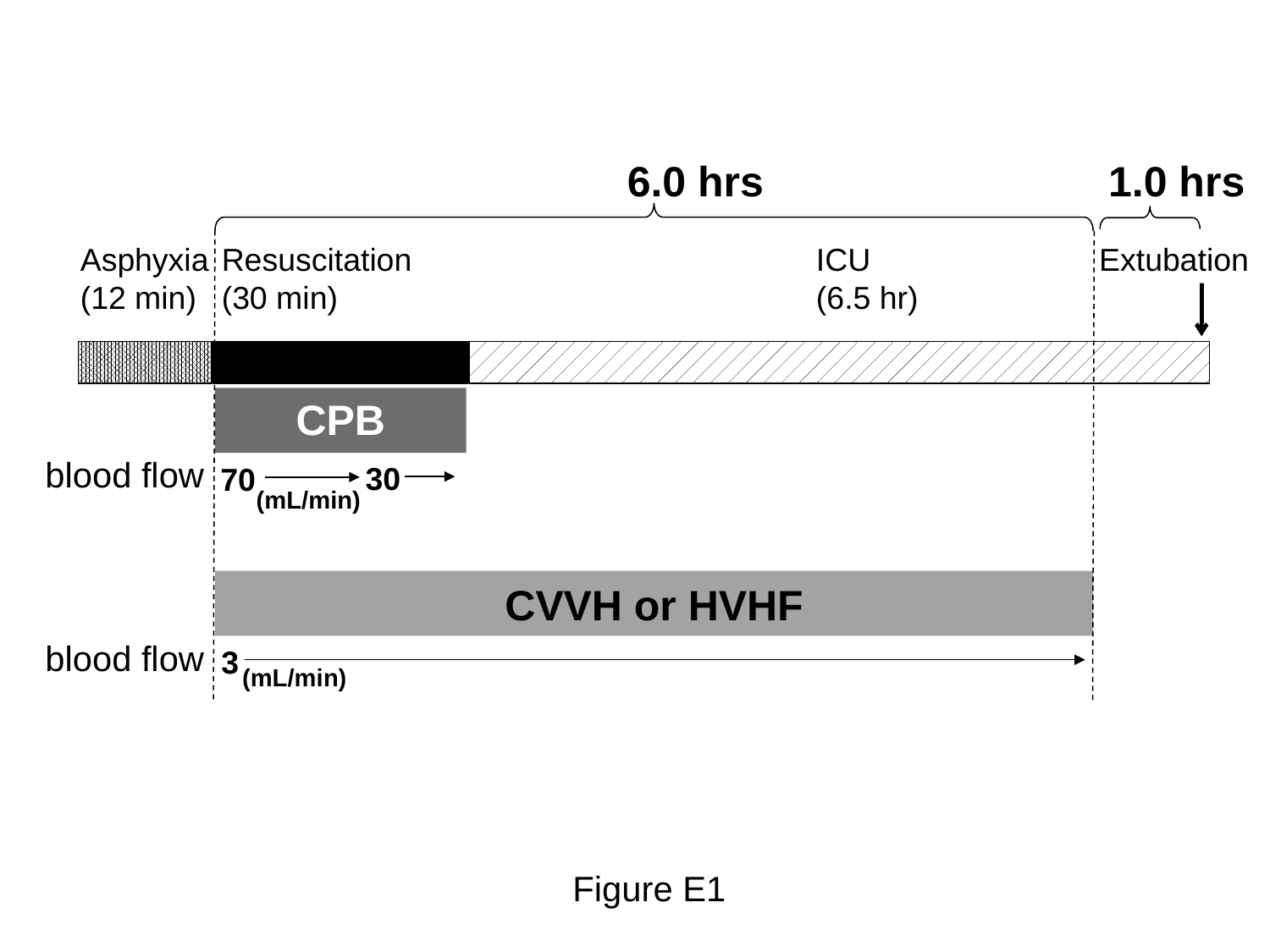

6.0 hrs
1.0 hrs
Asphyxia
(12 min)
Resuscitation
(30 min)
ICU
(6.5 hr)
Extubation
CPB
blood flow
30
70
(mL/min)
CVVH or HVHF
blood flow
3
(mL/min)
Figure E1
