# Supplementary figures and images for: The effects of early high-volume hemofiltration on prolonged cardiac arrest in rats with reperfusion by cardiopulmonary bypass: a randomized controlled animal study
Source: Intensive Care Med Exp. 2016 Sep 9;4(1):25. doi: 10.1186/s40635-016-0101-6 (PMC5017966; doi:10.1186/s40635-016-0101-6)

## Slide 1
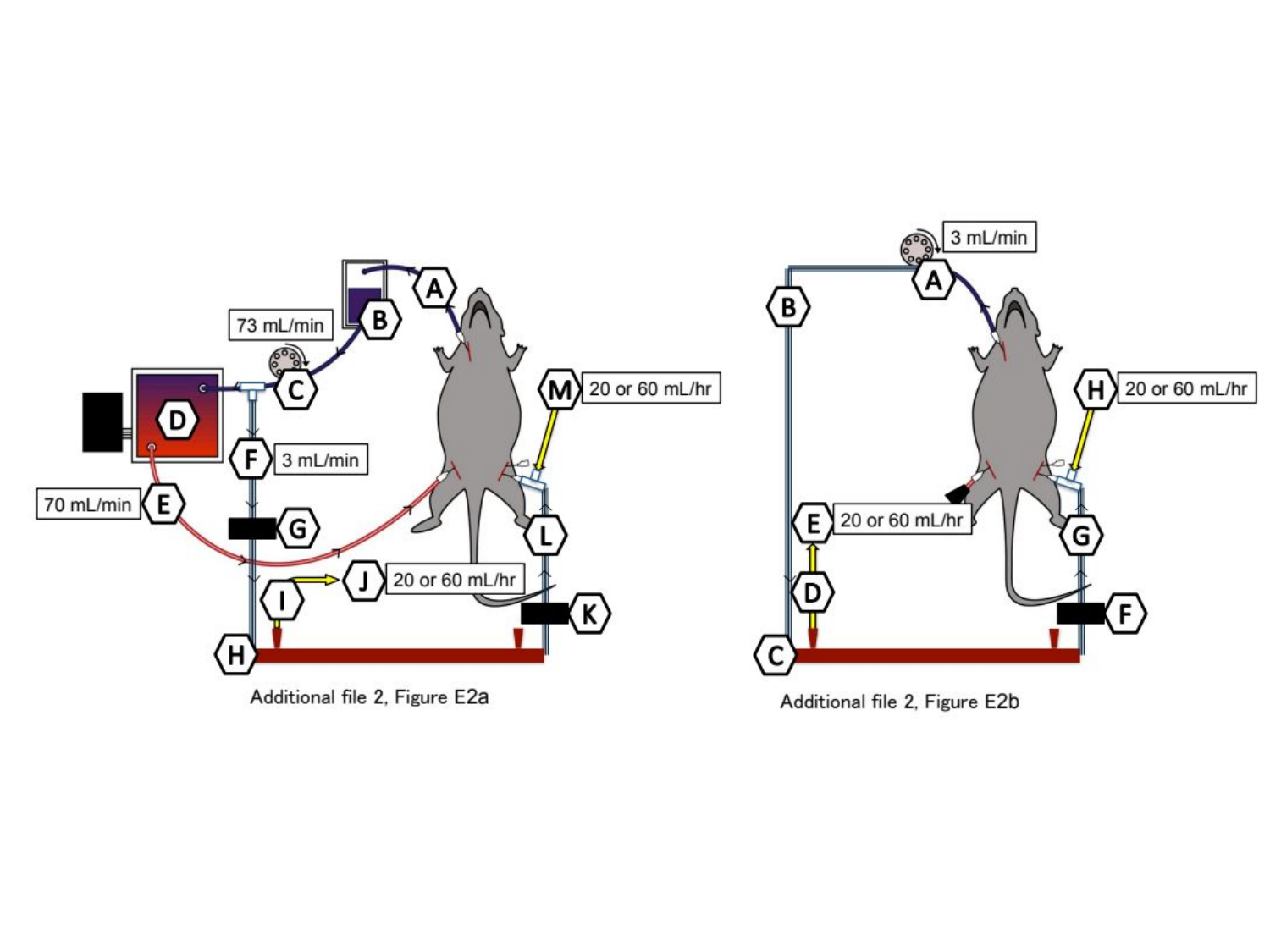

Supplement: Additional file 2: Figure E2a. — Schematic of the rodent continuous veno-venous hemofiltration circuit combined with emergency cardiopulmonary bypass circuit. The letters indicate the location of monitoring and circuit components; A, out flow tube for two extra corporeal circuits; B, venous reservoir; C, roller pump; D, oxygenator of cardiopulmonary bypass; E, inflow tube for cardiopulmonary bypass; F, outflow for continuous venovenous hemofiltration; G, flow sensor; H, pressure sensor at pre-filter; I, pressure sensor at side hole; J, filtration (effluent) pump; K, screw clamp; L, inflow tube for continuous venovenous hemofiltration; M, replacement infusion pump. Figure E2b. Schematic of the rodent continuous veno-venous hemofiltration circuit. The letters indicate the location of monitoring and circuit components; A, roller pump; B, outflow for continuous venovenous hemofiltration; C, pressure sensor at pre-filter; D, pressure sensor at side hole; E, filtration (effluent) pump; F, screw clamp; G, inflow tube for continuous venovenous hemofiltration; H, replacement infusion pump.Flow rates demonstrated in schematic were the typical settings of two extra corporeal circuits. Filtration and replacement fluid rates in the CVVH group were 20 mL/h and those in the HVHF group were 60 mL/h. (PPTX 563 kb) [file 40635_2016_101_MOESM2_ESM.pptx]

## Slide 1
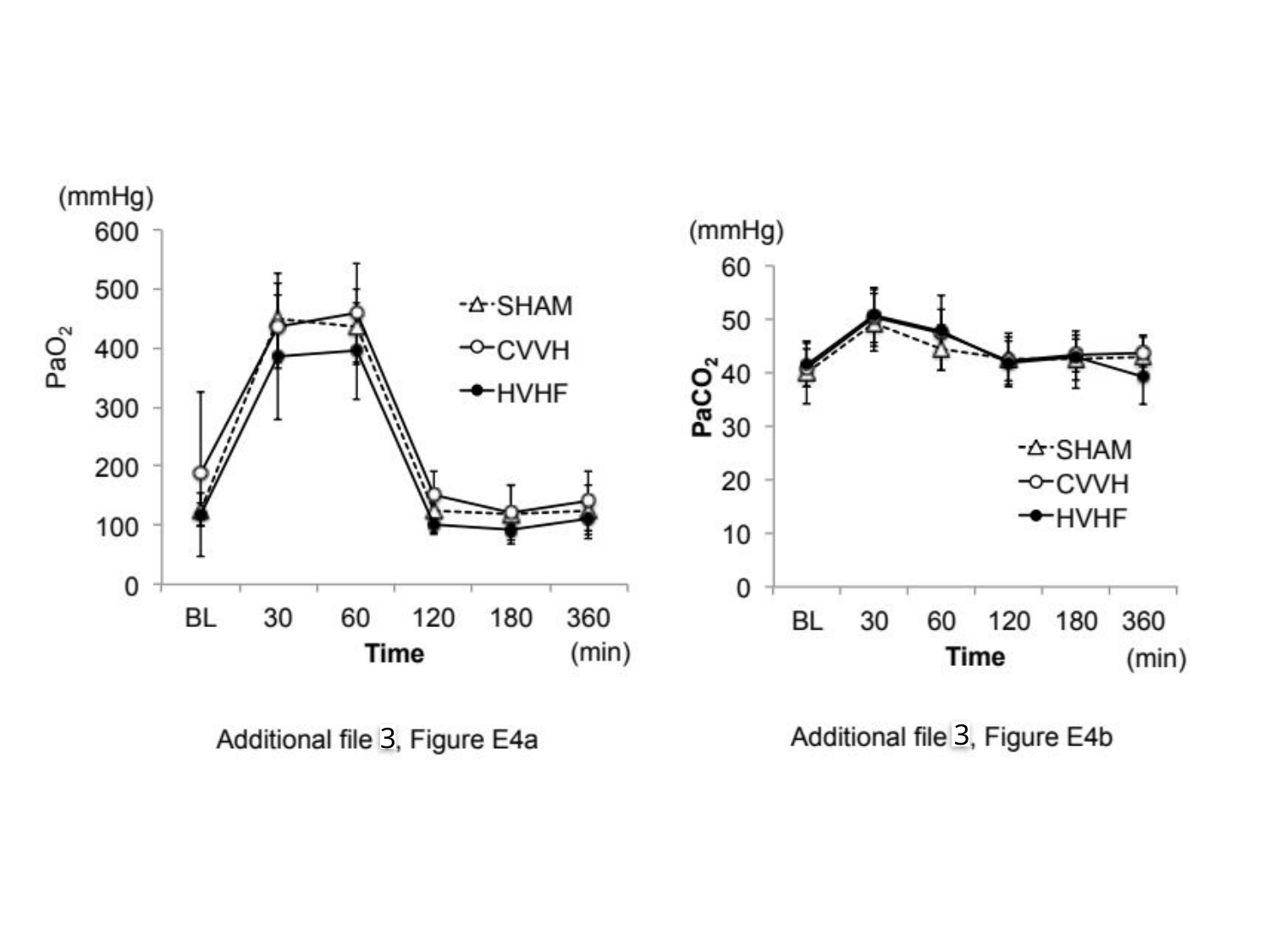

3
3

Supplement: Additional file 3: Figure E4 a and b. — Blood gas analysis (PaO2 and PaCO2) as a function of time compared between the three experimental groups. There were no differences between the three groups. (PPTX 131 kb) [file 40635_2016_101_MOESM3_ESM.pptx]
