# Supplementary material for: The effects of early high-volume hemofiltration on prolonged cardiac arrest in rats with reperfusion by cardiopulmonary bypass: a randomized controlled animal study
Source: Intensive Care Med Exp. 2016 Sep 9;4(1):25. doi: 10.1186/s40635-016-0101-6 (PMC5017966; doi:10.1186/s40635-016-0101-6)
Supplement: Additional file 4: — Additional manuscript. The contents include methods and results of "Estimated Blood Levels of IL-6 during CVVH Operation", "Blood Gas Analysis", "Table E1. Basal characteristics and resuscitation data of randomized animals" and "Table E2. Efficacy of hemofilter on IL-6 removal from circulating blood". (DOCX 305 kb) [file 40635_2016_101_MOESM4_ESM.docx]

Additional file 4

The Effects of Early High-Volume Hemofiltration on Prolonged Cardiac Arrest in Rats with Reperfusion by Cardiopulmonary Bypass

Koichiro Shinozaki MD, PhD^a, b^, Joshua W. Lampe, PhD^a^, Junhwan Kim, PhD^a^, Tai Yin, MD, PhD^a^, Tong Da, MD, PhD^a^, Shigeto Oda, MD, PhD^b^, Hiroyuki Hirasawa, MD, PhD^b^, and Lance B. Becker, MD, FAHA^a^

**MATERIALS AND METHODS**

**Modeling to Estimate Blood IL-6 levels during Hemofiltration**

The following general mass balance expression was used;

 (1)

There is no input in our animal models. Assuming no consumption, equation (1) can be rewritten:

 (2)

 (3)

Using discretization, the following equation is derived from the equation (2) and (3):


 (4)

Where t (min) is time from the start of CVVH operation. In this condition, “Output” is equal to an amount of molecules that were removed from the blood by CVVH. We find that:

 (5)

and:

 (6)

where C_b_ (pg/mL) is the concentration of IL-6 in the blood, V (mL/kg) is the total body water of a rat, SC is sieving coefficient of IL-6, and Q_f_ (mL/kg/min) is the hemofiltration rate. We used a sieving coefficient of 0.3 based on the results of our pilot study (Table E2). We assumed that the body was 60% water by weight [1]. We estimated C_g_(t) using a two parameter logistic curve fitted to mean blood concentrations of sham animals at each time point (baseline, 30, 60, 120, 180, and 360 mins after resuscitation). Combining (4), (5), and (6), we find:

 (7)

**Blood Gas Analysis**

Blood was sampled from the catheter placed in the femoral artery and blood gas analysis was performed at baseline, 30, 60, 120, 180, and 360 minutes after resuscitation.

**Statistical Analysis**

R square value for an estimate of logistic curve was calculated.

**RESULTS**

**Estimated Blood Levels of IL-6 during CVVH Operation**

The two parameter logistic curve was fitted to the mean blood IL-6 concentrations in the sham group animals (R square; 0.809, p=0.015).

 (8)

Using equation (6) and (8) as the IL-6 generation form, we are able to predict IL-6 removal based on equation (7), and an initial concentration of 0 pg/mL, as shown in Figure E3. Q_f_ was set at the same hemofiltration volume of our study groups (sham (0 mL/kg/hr); CVVH (40 mL/kg/hr); HVHF (120 mL/kg/hr)) and the highest volumes in the previous reports (300 mL/kg/hr) [2]. Assuming no extra generation of cytokine relating to the CVVH operation, Figure E3 shows IL-6 blood levels are reduced by hemofiltration over the 6 hr study period, however its effect is limited to a 16% decrease (HVHF) or a 6% decrease (CVVH). If hemofiltration is performed at the maximum rate of 300 mL/kg/hr, the estimated blood levels of IL-6 can be reduced as much as a 36%.

**Blood Gas Analysis**

The results of blood gas analysis are shown in Figure E4a and b. There were no differences in PaO_2_ or PaCO_2_ between the three groups at any time point.

**REFERENCES**

1. Foy JM, Schnieden H. Estimation of total body water (virtual tritium space) in the rat, cat, rabbit, guinea-pig and man, and of the biological half-life of tritium in man. J Physiol. 1960;154:169-176.

2. Nagashima M, Shin'oka T, Nollert G, Shum-Tim D, Rader CM, Mayer JE Jr. High-volume continuous hemofiltration during cardiopulmonary bypass attenuates pulmonary dysfunction in neonatal lambs after deep hypothermic circulatory arrest. Circulation. 1998;98:II378-84.

**Table E1.** Basal characteristics and resuscitation data of randomized animals

|  | Sham  n=8 | Continuous Veno-venous Hemofiltration | |
| --- | --- | --- | --- |
| Characteristic |  | Standard volume  Hemofiltration  n=8 | High volume  Hemofiltration  n=8 |
| Weight, g | 485±17 | 481±17 | 489±33 |
| Hematocrit, % | 42±2 | 43±1 | 42±1 |
| pH | 7.45±0.03 | 7.46±0.05 | 7.45±0.03 |
| pO_2_, mmHg | 126±29 | 135±26 | 119±21 |
| pCO_2_, mmHg | 40±6 | 41±4 | 42±4 |
| Na, mmol/L | 137±1 | 137±2 | 138±2 |
| K, mmol/L | 4.4±0.4 | 4.5±0.5 | 4.5±0.4 |
| BUN, mg/dL | 26±3 | 27±3 | 26±1 |
| Blood glucose, mg/dL | 319±84 | 355±69 | 294±70 |
| Lactate, mmol/L | 1.7±0.5 | 1.7±0.4 | 1.4±0.3 |
| Asphyxia to CA time, sec | 216±27 | 224±14 | 217±23 |
| Start ECPB to ROSC time, sec | 39 (32, 107) | 44 (36, 98) | 47 (36, 150) |

BUN indicates blood urea nitrogen; CA, cardiac arrest; CPB, cardiopulmonary bypass; ROSC, return of spontaneous circulation.

Blood samples were obtained from arterial line before asphyxia (base line).

CA was defined to occur when mean arterial pressure dropped below 20 mmHg. ROSC time was defined as the first time when spontaneous pulse pressure appeared.

Values are expressed as mean±SD and median (inter-quartile range).

**Table E2.** Efficacy of hemofilter on IL-6 removal from circulating blood

|  | Artery | Pre-filter | Post-filter | Effluent | SC | Differential | Differentialrates |
| --- | --- | --- | --- | --- | --- | --- | --- |
| Rat1 |  |  |  |  |  |  |  |
| T_1_, pg/mL | 0 | 0 | 0 | 0 | N/A | 0 | N/A |
| T_2_, pg/mL | 1652 | 1537 | 763 | 622 | 0.54 | 774 | 50.3 (%) |
| T_3_, pg/mL | 2967 | 3932 | 2483 | 720 | 0.22 | 1449 | 36.8 (%) |
| T_4_, pg/mL | 7958 | 6640 | 5838 | 1787 | 0.29 | 802 | 12.1 (%) |
|  |  |  |  |  |  |  |  |
| Rat2 |  |  |  |  |  |  |  |
| T_1_, pg/mL | 0 | 0 | 0 | 0 | N/A | 0 | N/A |
| T_2_, pg/mL | 11459 | 11560 | 8699 | 518 | 0.05 | 2862 | 24.8 (%) |
| T_3_, pg/mL | 8575 | 8712 | 4437 | 1276 | 0.19 | 4276 | 49.1 (%) |
| T_4_, pg/mL | 17805 | 16579 | 13147 | 5514 | 0.37 | 3432 | 20.7 (%) |

Sieving coefficient (SC) is calculated as:

$$SC=\frac{2Cf}{Ci+Co}$$

Where Cf is a concentration of the effluent sample, Ci is a concentration of the blood collected at the pre-filter port and Co is a concentration of the blood collected at the post-filter port.

Differential is calculated as:

$$\text{Differential = }Co-Ci$$

Differential rate is calculated as:

$$\text{Differential rate = }\frac{Co-Ci}{Ci}$$

 indicates negative quantity;, positive quantity; N/A, not applicable.

Four blood samples (artery, pre, post-filter, and effluent) were collected within 5 min at all time points (T_1_ to T_4_).

**FIGURE LEGENDS**

Figure E1. Experimental Protocol

Flow rates shown in this figure were typical of our experiments.

ICU indicates intensive care unit; CPB, cardiopulmonary bypass; CVVH, continuous veno-venous hemofiltration.

Figure E2a. Schematic of the rodent continuous veno-venous hemofiltration circuit combined with emergency cardiopulmonary bypass circuit

The letters indicate the location of monitoring and circuit components; A, out flow tube for two extra corporeal circuits; B, venous reservoir; C, roller pump; D, oxygenator of cardiopulmonary bypass; E, inflow tube for cardiopulmonary bypass; F, outflow for continuous venovenous hemofiltration; G, flow sensor; H, pressure sensor at pre-filter; I, pressure sensor at side hole; J, filtration (effluent) pump; K, screw clamp; L, inflow tube for continuous venovenous hemofiltration; M, replacement infusion pump.

Figure E2b. Schematic of the rodent continuous veno-venous hemofiltration circuit

The letters indicate the location of monitoring and circuit components; A, roller pump; B, outflow for continuous venovenous hemofiltration; C, pressure sensor at pre-filter; D, pressure sensor at side hole; E, filtration (effluent) pump; F, screw clamp; G, inflow tube for continuous venovenous hemofiltration; H, replacement infusion pump.

Flow rates demonstrated in schematic were the typical settings of two extra corporeal circuits. Filtration and replacement fluid rates in the CVVH group were 20 mL/h and those in the HVHF group were 60 mL/h.

Figure E3. Predicted blood levels of IL-6 compared between differing settings of hemofiltration

This estimate was based on mean blood levels of sham animals in this study. The sigmoid trend curve was generated by fitting the two-parameter logistic curve to our data. Sieving coefficient of our investigated hemofilter was set at 0.3. We assumed that a total volume of 600 mL was purified using CVVH in our model. This number was made by a calculation of 1000 g × 60%, where 1000 g was an unit weight (kg) and 60% was a rate of body water contained in the animal body.

Figure E4a and b. Blood gas analysis (PaO and PaCO ) as a function of time compared between the three experimental groups. There were no differences between the three groups.
