# Supplementary material for: The effects of early high-volume hemofiltration on prolonged cardiac arrest in rats with reperfusion by cardiopulmonary bypass: a randomized controlled animal study
Source: Intensive Care Med Exp. 2016 Sep 9;4(1):25. doi: 10.1186/s40635-016-0101-6 (PMC5017966; doi:10.1186/s40635-016-0101-6)
Supplement: Additional file 5: Figure E3. — Predicted blood levels of IL-6 compared between differing settings of hemofiltration. This estimate was based on mean blood levels of sham animals in this study. The sigmoid trend curve was generated by fitting the two-parameter logistic curve to our data. Sieving coefficient of our investigated hemofilter was set at 0.3. We assumed that a total volume of 600 mL was purified using CVVH in our model. This number was made by a calculation of 1000 g × 60 %, where 1000 g was an unit weight (kg) and 60 % was a rate of body water contained in the animal body. (PPTX 93.4 kb) [file 40635_2016_101_MOESM5_ESM.pptx]

## Slide 1
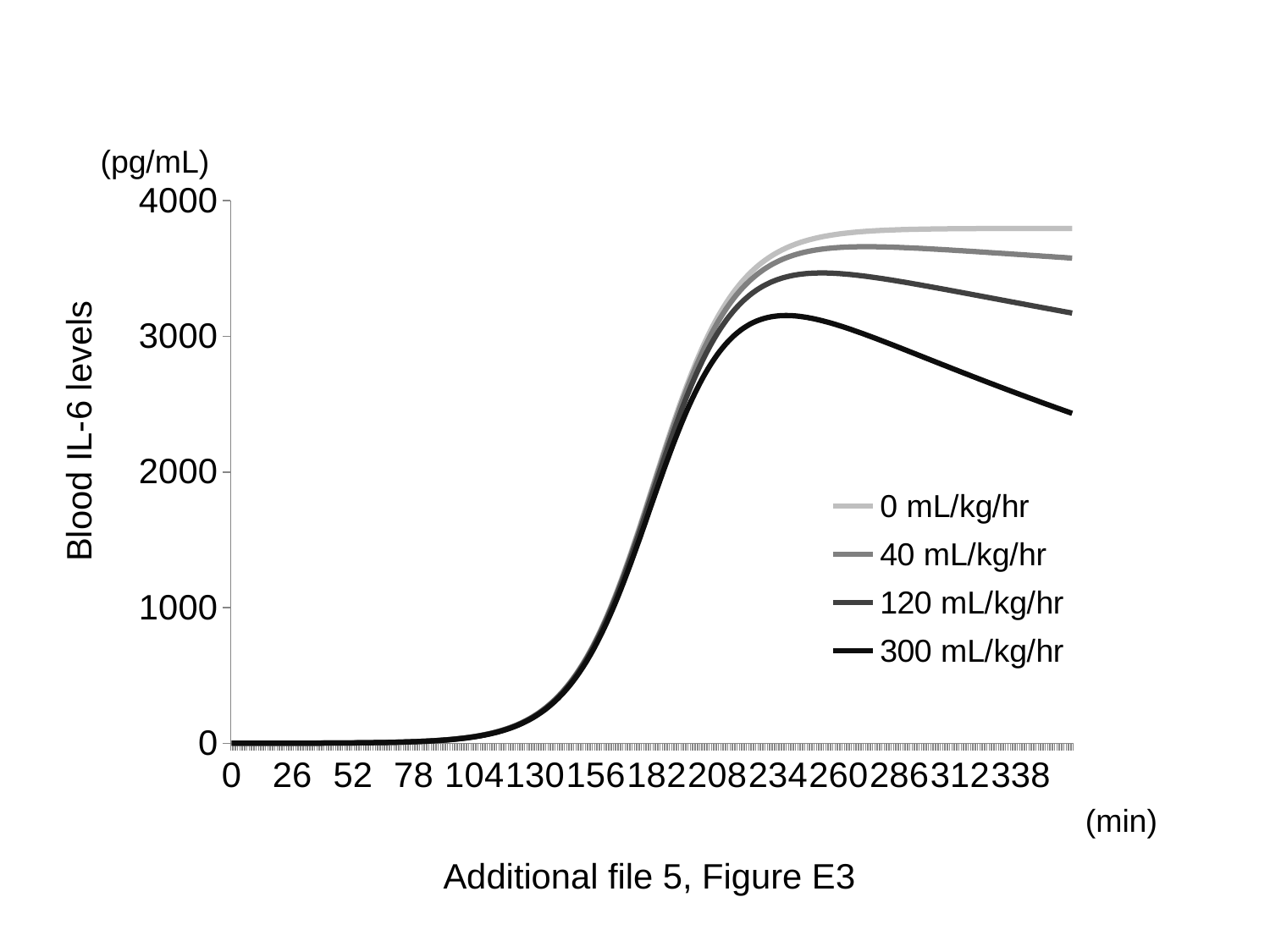

(pg/mL)
### Chart
| Category | 0 mL/kg/hr | 40 mL/kg/hr | 120 mL/kg/hr | 300 mL/kg/hr |
|---|---|---|---|---|
| 0 | 0.140090094611742 | 0.0 | 0.0 | 0.0 |
| 1 | 0.148243167951563 | 0.00815038282561846 | 0.00814492026648078 | 0.0081530733398206 |
| 2 | 0.156870720646752 | 0.0167723988020854 | 0.0167557004887079 | 0.0167602433516598 |
| 3 | 0.166000363329334 | 0.0258934938109782 | 0.0258594578281193 | 0.0258479854258634 |
| 4 | 0.175661313052128 | 0.0355427105674058 | 0.0354848871433621 | 0.0354443151850925 |
| 5 | 0.185884486718591 | 0.0457507814920711 | 0.0456623527490148 | 0.0455788780635923 |
| 6 | 0.196702599942538 | 0.0565502269807617 | 0.0564239855069887 | 0.0562830440923802 |
| 7 | 0.20815027165281 | 0.0679754593844664 | 0.0678037855600442 | 0.0675900081924219 |
| 8 | 0.220264134776204 | 0.0800628930314329 | 0.0798377310347548 | 0.079534896295335 |
| 9 | 0.233082953351242 | 0.0928510606416408 | 0.0925638930601831 | 0.0921548776296348 |
| 10 | 0.246647746445752 | 0.106380736504418 | 0.106022557468538 | 0.105489283530071 |
| 11 | 0.261001919272762 | 0.120695066811349 | 0.120256353565253 | 0.119579733148256 |
| 12 | 0.276191401922011 | 0.135839707559275 | 0.135310390378287 | 0.134470266464634 |
| 13 | 0.292264796148451 | 0.151862970462126 | 0.151232400820122 | 0.150207485024912 |
| 14 | 0.309273530684604 | 0.168815977335629 | 0.168072894220919 | 0.166840700848502 |
| 15 | 0.327272025570536 | 0.186752823445728 | 0.185885317717744 | 0.184422093982313 |
| 16 | 0.346317866023699 | 0.205730750339804 | 0.204726227012736 | 0.20300687920052 |
| 17 | 0.366471986400936 | 0.225810328709705 | 0.224655467042583 | 0.222653482379756 |
| 18 | 0.387798864836791 | 0.247055651867202 | 0.24573636313296 | 0.243423727109662 |
| 19 | 0.410366729175828 | 0.269534540445891 | 0.268035923244525 | 0.265383032130925 |
| 20 | 0.434247774852197 | 0.293318758978839 | 0.291625051951973 | 0.288600620226966 |
| 21 | 0.459518395407223 | 0.31848424503862 | 0.316578776834492 | 0.313149739231426 |
| 22 | 0.486259426375437 | 0.345111351665751 | 0.342976487994903 | 0.339107895851561 |
| 23 | 0.51455640331136 | 0.373285103853236 | 0.370902191465895 | 0.366557103047854 |
| 24 | 0.544499834773644 | 0.403095469898865 | 0.400444777305251 | 0.395584141752519 |
| 25 | 0.576185491129898 | 0.434637648483455 | 0.431698303227844 | 0.426280837754391 |
| 26 | 0.609714710094937 | 0.468012372382236 | 0.46476229467069 | 0.458744354625044 |
| 27 | 0.645194719967339 | 0.503326229768495 | 0.499742062238549 | 0.493077503610885 |
| 28 | 0.682738981584288 | 0.540692004123286 | 0.536749037531642 | 0.529389071468806 |
| 29 | 0.722467550072812 | 0.580229033822848 | 0.575901128414146 | 0.567794167278658 |
| 30 | 0.764507457536965 | 0.622063592536376 | 0.617323094842421 | 0.608414589324614 |
| 31 | 0.808993117885263 | 0.666329291631223 | 0.661146946435529 | 0.651379213199601 |
| 32 | 0.856066755071121 | 0.713167505850571 | 0.707512363037765 | 0.69682440235246 |
| 33 | 0.905878856091199 | 0.762727823600382 | 0.756567139593785 | 0.744894442366657 |
| 34 | 0.95858865016273 | 0.815168523258081 | 0.808467656731651 | 0.795742000332271 |
| 35 | 1.014364615581232 | 0.870657076995319 | 0.863379378528002 | 0.849528610749942 |
| 36 | 1.073385015844724 | 0.929370683691317 | 0.921477379012704 | 0.90642518948656 |
| 37 | 1.135838466719936 | 0.991496832602122 | 0.982946899058028 | 0.966612577388056 |
| 38 | 1.201924536020138 | 1.057233899544697 | 1.047983935389872 | 1.030282115244788 |
| 39 | 1.271854377963542 | 1.126791777453409 | 1.116795863555942 | 1.097636251900079 |
| 40 | 1.345851404085778 | 1.200392543270465 | 1.1896020967885 | 1.168889187392565 |
| 41 | 1.4241519927902 | 1.278271163241336 | 1.266634782807428 | 1.244267553128506 |
| 42 | 1.507006239735764 | 1.360676238801538 | 1.34813954072324 | 1.32401113119125 |
| 43 | 1.594678751384465 | 1.44787079536259 | 1.43437624031957 | 1.408373615011972 |
| 44 | 1.687449484158883 | 1.540133116432723 | 1.525619826120893 | 1.497623413748859 |
| 45 | 1.78561463179571 | 1.637757625642407 | 1.622161188783962 | 1.592044502851315 |
| 46 | 1.889487563623448 | 1.74105581938618 | 1.724308086491088 | 1.691937323421924 |
| 47 | 1.999399816642066 | 1.850357252940905 | 1.832386119170197 | 1.797619733131988 |
| 48 | 2.115702144439679 | 1.966010583076874 | 1.946739758520842 | 1.90942801159677 |
| 49 | 2.23876562614644 | 2.088384670342256 | 2.067733436987375 | 2.02771792327454 |
| 50 | 2.368982838799328 | 2.217869744373755 | 2.195752698990624 | 2.152865841119238 |
| 51 | 2.506769096673426 | 2.354878635767112 | 2.331205417907856 | 2.28526993439054 |
| 52 | 2.652563761326268 | 2.499848078230817 | 2.47452308247814 | 2.425351424207407 |
| 53 | 2.806831626301824 | 2.653240084945114 | 2.626162156506243 | 2.573555910622444 |
| 54 | 2.970064380650408 | 2.815543403256717 | 2.786605515943972 | 2.730354775194471 |
| 55 | 3.142782155640144 | 2.987275052057646 | 2.956363967642774 | 2.896246663246208 |
| 56 | 3.32553515926522 | 3.168981946424346 | 3.135977854296582 | 3.071759050213182 |
| 57 | 3.518905403396086 | 3.361242614332331 | 3.326018750329021 | 3.257449896718516 |
| 58 | 3.723508528667347 | 3.564669010509522 | 3.527091253724683 | 3.453909397247981 |
| 59 | 3.939995732460528 | 3.779908432751982 | 3.739834879060346 | 3.66176182754804 |
| 60 | 4.169055805611475 | 4.007645546295969 | 3.964926057259091 | 3.881667496130122 |
| 61 | 4.411417283755976 | 4.248604522122418 | 4.203080247868177 | 4.114324805534283 |
| 62 | 4.667850719522031 | 4.503551295362385 | 4.455054169950616 | 4.360472429286529 |
| 63 | 4.93917108208439 | 4.773295950277614 | 4.721648157980447 | 4.620891610775645 |
| 64 | 5.226240290913898 | 5.058695238604614 | 5.003708649443141 | 4.896408590578223 |
| 65 | 5.52996989088468 | 5.360655238378666 | 5.302130811164503 | 5.187897169072555 |
| 66 | 5.851323876241508 | 5.680134160691666 | 5.617861311724822 | 5.496281411506712 |
| 67 | 6.191321671281698 | 6.018145312186448 | 5.951901247658243 | 5.822538503018133 |
| 68 | 6.551041275967226 | 6.375760221449424 | 6.305309231491431 | 6.167701761446103 |
| 69 | 6.93162258505474 | 6.754111937831845 | 6.679204650038365 | 6.532863816130017 |
| 70 | 7.334270889711774 | 7.15439851160887 | 7.074771101740703 | 6.919179961246724 |
| 71 | 7.760260570976905 | 7.577886664770355 | 7.49326002222283 | 7.327871692608737 |
| 72 | 8.210938994818008 | 8.025915662132219 | 7.935994507617869 | 7.760230437218321 |
| 73 | 8.68773061894526 | 8.499901392855005 | 8.404373345613344 | 8.217621485252469 |
| 74 | 9.192141321942145 | 9.001340672860257 | 8.89987526456165 | 8.70148813453628 |
| 75 | 9.725762965687062 | 9.531815779040693 | 9.424063411398258 | 9.213356057944857 |
| 76 | 10.29027820244715 | 10.09299922656556 | 9.978590069510185 | 9.754837904560082 |
| 77 | 10.88746553843292 | 10.6866588009857 | 10.56520162809046 | 10.32763814578445 |
| 78 | 11.51920466600338 | 11.31466285723972 | 11.18574381490526 | 10.93355817799045 |
| 79 | 12.18748207710324 | 11.97898589805104 | 11.84216720477912 | 11.57450169364535 |
| 80 | 12.89439697089254 | 12.68171444457903 | 12.53653301646984 | 12.25248033320053 |
| 81 | 13.64216746888936 | 13.4250532125448 | 13.2710192109522 | 12.96961963036435 |
| 82 | 14.43313715128357 | 14.21133160738368 | 14.04792690445306 | 13.72816526368265 |
| 83 | 15.26978192838635 | 15.04301055227958 | 14.8696871098743 | 14.53048962762623 |
| 84 | 16.15471726145072 | 15.9226896632018 | 15.73886782049572 | 15.37909873662153 |
| 85 | 17.09070574732351 | 16.85311478528539 | 16.65818145006215 | 16.27663947565277 |
| 86 | 18.08066508156057 | 17.837185905063 | 17.6304926435149 | 17.22590721120068 |
| 87 | 19.1276764147436 | 18.8779654531574 | 18.65882647272122 | 18.22985377635571 |
| 88 | 20.23499311676846 | 19.97868701207105 | 19.74637703157121 | 19.29159584393968 |
| 89 | 21.40604996381625 | 21.14276444364534 | 20.89651644474052 | 20.41442370137763 |
| 90 | 22.64447276255756 | 22.37380145059665 | 22.11280430423835 | 21.6018104408655 |
| 91 | 23.95408842585856 | 23.67560158625007 | 23.39899754757181 | 22.85742157806433 |
| 92 | 25.33893551383906 | 25.05217872616808 | 24.75906079091676 | 24.18512511209967 |
| 93 | 26.80327525355473 | 26.50776801479001 | 26.1971771301018 | 25.58900203903508 |
| 94 | 28.35160304981864 | 28.04683729943627 | 27.71775942143934 | 27.07335733020141 |
| 95 | 29.98866049871392 | 29.6740990630646 | 29.32546205346429 | 28.64273138577104 |
| 96 | 31.71944791415337 | 31.39452286596615 | 31.02519321943484 | 30.30191197274621 |
| 97 | 33.54923737638412 | 33.21334830512858 | 32.82212769898392 | 32.05594665504495 |
| 98 | 35.4835863095797 | 35.1360984982355 | 34.72172015554731 | 33.91015572160306 |
| 99 | 37.5283515935764 | 37.16859409718406 | 36.7297189541045 | 35.87014561629574 |
| 100 | 39.68970421235052 | 39.31696783354192 | 38.85218050130571 | 37.94182287102911 |
| 101 | 41.97414443896064 | 41.58767959549196 | 41.09548410718792 | 40.13140854046166 |
| 102 | 44.38851755334132 | 43.98753203247881 | 43.46634736434719 | 42.44545313349138 |
| 103 | 46.94003008548881 | 46.52368667991978 | 45.97184203659805 | 44.89085203280496 |
| 104 | 49.63626657215698 | 49.20368059194284 | 48.61941044474295 | 47.47486138939094 |
| 105 | 52.48520681015107 | 52.03544346506305 | 51.4168823320543 | 50.20511447391172 |
| 106 | 55.49524358356275 | 55.0273152299962 | 54.37249218636051 | 53.08963846113848 |
| 107 | 58.675200835816 | 58.18806408233029 | 57.49489698917514 | 56.136871617239 |
| 108 | 62.03435225007747 | 61.52690491547788 | 60.79319435503317 | 59.35568085245725 |
| 109 | 65.58244019337891 | 65.05351811113593 | 64.27694101603613 | 62.75537959362786 |
| 110 | 69.32969497061654 | 68.77806863332023 | 67.9561715974803 | 66.34574592188123 |
| 111 | 73.28685432435117 | 72.7112253618193 | 71.84141762026405 | 70.13704091081115 |
| 112 | 77.4651831049723 | 76.86418058957342 | 75.94372665448431 | 74.14002708915525 |
| 113 | 81.8764930231913 | 81.2486695959251 | 80.27468153613063 | 78.36598693965124 |
| 114 | 86.53316238295899 | 85.87699019383703 | 84.8464195450025 | 82.82674133206939 |
| 115 | 91.4481556776402 | 90.76202213396708 | 89.67165142684385 | 87.53466777342095 |
| 116 | 96.6350429155879 | 95.9172462318226 | 94.76368012612721 | 92.50271834193556 |
| 117 | 102.1080185230382 | 101.3567630660655 | 100.1364190778436 | 97.74443715353048 |
| 118 | 107.8819196524589 | 107.0953110763016 | 105.8044098870569 | 103.2739771900675 |
| 119 | 113.972243703106 | 113.1482838673567 | 111.7828392037662 | 109.1061162977393 |
| 120 | 120.3951648374718 | 119.5317465040721 | 118.0875545777941 | 115.2562721413609 |
| 121 | 127.1675492526412 | 126.2624505560381 | 124.7350790539705 | 121.740515876177 |
| 122 | 134.3069689392372 | 133.357847625454 | 131.7426242418258 | 128.5755842730823 |
| 123 | 141.8317136327528 | 140.8361010635044 | 139.1281015664061 | 135.7788900059153 |
| 124 | 149.7608006326761 | 148.7160955513666 | 146.9101313777631 | 143.3685297808238 |
| 125 | 158.1139821340981 | 157.0174441913613 | 155.108049566306 | 151.3632899577937 |
| 126 | 166.9117496846076 | 165.7604927219961 | 163.7419112996986 | 159.7826492834087 |
| 127 | 176.1753353465087 | 174.9663204380304 | 172.832491464638 | 168.6467783221012 |
| 128 | 185.9267091110481 | 184.656737363483 | 182.4012813639482 | 177.9765351408354 |
| 129 | 196.1885720778483 | 194.8542771921742 | 192.4704811864176 | 187.7934567697835 |
| 130 | 206.984344879592 | 205.58218547742 | 203.0629877341732 | 198.1197459296029 |
| 131 | 218.3381507998193 | 216.8644025204861 | 214.2023768607461 | 208.9782524850062 |
| 132 | 230.2747930011906 | 228.7255403770992 | 225.9128800430553 | 220.392449055165 |
| 133 | 242.8197252535958 | 241.1908533735366 | 238.2193544831648 | 232.3864001849322 |
| 134 | 255.9990155270096 | 254.2862014995469 | 251.1472461118223 | 244.9847244578837 |
| 135 | 269.8393017941441 | 268.0380060257184 | 264.7225448465769 | 258.2125489138735 |
| 136 | 284.3677393739642 | 282.4731966791485 | 278.9717314439699 | 272.0954551214088 |
| 137 | 299.6119391404242 | 297.6191497047818 | 293.9217152792197 | 286.6594162500656 |
| 138 | 315.5998959228879 | 313.5036161421039 | 309.5997623896231 | 301.9307244919036 |
| 139 | 332.359906437256 | 330.154639659676 | 326.0334131310869 | 317.9359081950422 |
| 140 | 349.9204761116607 | 347.6004633150003 | 343.2503888226863 | 334.7016380989592 |
| 141 | 368.3102142095132 | 365.8694246463867 | 361.2784867936182 | 352.2546221015645 |
| 142 | 387.5577167076618 | 384.9898385585763 | 380.1454633024751 | 370.6214880444593 |
| 143 | 407.6914364603546 | 404.9898675370277 | 399.8789038721125 | 389.8286540770408 |
| 144 | 428.7395402724806 | 425.8973788186086 | 420.5060806765546 | 409.9021862539742 |
| 145 | 450.7297526199787 | 447.7397882610217 | 442.0537967310283 | 430.8676431358377 |
| 146 | 473.6891858929043 | 470.543890790841 | 464.5482167739499 | 452.7499073009236 |
| 147 | 497.6441571987872 | 494.3356774722316 | 488.0146848917528 | 475.5730038385541 |
| 148 | 522.619991951414 | 519.1401394258244 | 512.4775291247328 | 499.3599060815847 |
| 149 | 548.6408146835404 | 544.9810590404384 | 537.959853505005 | 524.1323290485072 |
| 150 | 575.729327760946 | 571.8807891590455 | 564.4833182158281 | 549.9105113032914 |
| 151 | 603.906578938787 | 599.8600211835717 | 592.0679088242726 | 576.712986202874 |
| 152 | 633.1917189874612 | 628.9375433290427 | 620.7316958240764 | 604.5563437860413 |
| 153 | 663.601750921329 | 659.1299905630736 | 650.4905860301867 | 633.4549848604429 |
| 154 | 695.1512726856304 | 690.4515880883074 | 681.3580676866939 | 663.4208691625944 |
| 155 | 727.8522154896514 | 722.913890557137 | 713.3449514802245 | 694.4632597937084 |
| 156 | 761.71358031117 | 756.525519544377 | 746.4591099854375 | 726.5884664657432 |
| 157 | 796.7411754307441 | 791.291902136112 | 780.7052183999077 | 759.7995904191532 |
| 158 | 832.9373581757964 | 827.2150138131534 | 816.0844997438174 | 794.0962741881573 |
| 159 | 870.3007843532761 | 864.2931291054364 | 852.5944779953758 | 829.4744596801665 |
| 160 | 908.8261691155857 | 902.5205837581731 | 890.2287428949304 | 865.9261582932772 |
| 161 | 948.5040632253756 | 941.887552370263 | 928.976730367713 | 903.4392370073323 |
| 162 | 989.3206488489386 | 982.3798456282897 | 968.8235226752865 | 941.997224538379 |
| 163 | 1031.257559103685 | 1023.978731353593 | 1009.749672497102 | 981.5791417317782 |
| 164 | 1074.291725600506 | 1066.660783594123 | 1051.73105515493 | 1022.15936037427 |
| 165 | 1118.39525814745 | 1110.39776391674 | 1094.738753114171 | 1063.707494520278 |
| 166 | 1163.53536060745 | 1155.156538880836 | 1138.738976718596 | 1106.188328243976 |
| 167 | 1209.674286624213 | 1200.899037394183 | 1183.693024832623 | 1149.561783440131 |
| 168 | 1256.769338542827 | 1247.582251263324 | 1229.557288674487 | 1193.782930900144 |
| 169 | 1304.772912354993 | 1295.158281753215 | 1276.283301624166 | 1238.80204738506 |
| 170 | 1353.632590896572 | 1343.574434367897 | 1323.81783718558 | 1284.564720808176 |
| 171 | 1403.291286824672 | 1392.773363363 | 1372.103056580565 | 1331.012004934255 |
| 172 | 1453.687436114546 | 1442.693266713698 | 1421.07670666457 | 1378.080624211794 |
| 173 | 1504.755241958774 | 1493.268131403982 | 1470.67236799629 | 1425.703228495492 |
| 174 | 1556.424968042045 | 1544.428027994282 | 1520.81975198548 | 1473.808696507524 |
| 175 | 1608.623279227267 | 1596.099452487575 | 1571.445045107531 | 1522.322485951477 |
| 176 | 1661.273626748088 | 1648.205712574393 | 1622.471297235724 | 1571.167027257419 |
| 177 | 1714.29667408641 | 1700.667354421942 | 1673.818850229471 | 1620.262157027597 |
| 178 | 1767.610758849032 | 1753.402625309634 | 1725.405802057102 | 1669.52558639765 |
| 179 | 1821.132385172464 | 1806.327966630027 | 1777.148500952153 | 1718.873398755089 |
| 180 | 1874.776740504928 | 1859.358531096243 | 1828.962063428332 | 1768.220570590665 |
| 181 | 1928.458230062581 | 1912.40871744708 | 1880.760909432998 | 1817.481508721841 |
| 182 | 1982.091021851817 | 1965.392715538269 | 1932.459307521013 | 1866.570596739272 |
| 183 | 2035.589594905377 | 2018.225054466594 | 1983.971922693998 | 1915.402743300984 |
| 184 | 2088.869283305467 | 2070.821146301537 | 2035.214359482994 | 1963.893924842821 |
| 185 | 2141.846808664769 | 2123.097818099194 | 2086.103692957456 | 2011.96171539002 |
| 186 | 2194.440794002698 | 2174.973825141984 | 2136.558980617086 | 2059.52579643947 |
| 187 | 2246.572252380084 | 2226.37033877581 | 2186.50174855548 | 2106.508440325743 |
| 188 | 2298.165044226072 | 2277.211402788693 | 2235.856445861067 | 2152.834961070931 |
| 189 | 2349.146297986427 | 2327.424352972386 | 2284.550861921799 | 2198.434127428608 |
| 190 | 2399.446789519904 | 2376.940195307176 | 2332.516502101821 | 2243.238533643514 |
| 191 | 2449.001276542396 | 2425.6939390845 | 2379.68891813519 | 2287.184924331899 |
| 192 | 2497.748785339913 | 2473.624882204218 | 2426.007990505777 | 2330.21447081859 |
| 193 | 2545.632847913287 | 2520.676846825811 | 2471.418161026067 | 2372.272997214913 |
| 194 | 2592.601688652609 | 2566.798364488237 | 2515.868614763624 | 2413.311155461198 |
| 195 | 2638.60836054306 | 2611.942810716684 | 2559.313411367422 | 2453.284549462982 |
| 196 | 2683.610831752881 | 2656.06848998347 | 2601.711566694665 | 2492.15380929916 |
| 197 | 2727.572024230551 | 2699.138672665928 | 2643.027086413163 | 2529.88461725358 |
| 198 | 2770.459806625946 | 2741.121586331152 | 2683.228953939749 | 2566.447688105839 |
| 199 | 2812.246944438599 | 2781.990364264838 | 2722.29107566065 | 2601.81870669823 |
| 200 | 2852.911010778268 | 2821.72295464241 | 2760.19218685832 | 2635.978226271153 |
| 201 | 2892.434261495745 | 2860.301994112116 | 2796.915722138218 | 2668.911531422951 |
| 202 | 2930.803478706134 | 2897.714649822768 | 2832.449654409259 | 2700.608469804783 |
| 203 | 2968.009786886566 | 2933.95243408706 | 2866.786306627102 | 2731.063256810704 |
| 204 | 3004.048445792228 | 2969.010995932038 | 2899.922140567234 | 2760.274257574342 |
| 205 | 3038.918624407635 | 3002.889893759842 | 2931.85752686346 | 2788.243750545812 |
| 206 | 3072.623160044515 | 3035.59235323502 | 2962.596500437836 | 2814.977676806326 |
| 207 | 3105.168306525213 | 3067.125014340812 | 2992.146505271615 | 2840.485379095008 |
| 208 | 3136.563475162744 | 3097.497671317963 | 3020.51813223524 | 2864.779334284804 |
| 209 | 3166.820971977776 | 3126.723008927508 | 3047.724853421219 | 2887.874882764122 |
| 210 | 3195.955734290118 | 3154.81633817534 | 3073.782756117827 | 2909.789957869553 |
| 211 | 3223.985069498968 | 3181.795334311973 | 3098.710279235351 | 2930.544818183727 |
| 212 | 3250.928398531433 | 3207.679779585534 | 3122.52795465955 | 2950.161785170734 |
| 213 | 3276.807006102185 | 3232.49131288853 | 3145.258155668071 | 2968.66498827856 |
| 214 | 3301.643799595411 | 3256.253188106644 | 3166.924854212135 | 2986.080119301088 |
| 215 | 3325.463078060125 | 3278.99004265739 | 3187.553388544173 | 3002.43419746755 |
| 216 | 3348.290312506233 | 3300.727677402054 | 3207.170242367291 | 3017.75534641999 |
| 217 | 3370.151938405403 | 3321.492848831135 | 3225.802836398194 | 3032.072583953111 |
| 218 | 3391.07516104049 | 3341.31307416264 | 3243.47933297425 | 3045.415625128315 |
| 219 | 3411.087774111747 | 3360.216449757108 | 3260.228454099459 | 3057.814699136751 |
| 220 | 3430.2179917982 | 3378.231483043305 | 3276.079313114125 | 3069.300380075361 |
| 221 | 3448.494294288466 | 3395.386937964346 | 3291.061259988787 | 3079.90343161544 |
| 222 | 3465.945286636586 | 3411.711693795462 | 3305.20374008457 | 3089.65466538452 |
| 223 | 3482.59957066399 | 3427.234617050186 | 3318.536166087862 | 3098.584812748463 |
| 224 | 3498.485629517366 | 3441.984446080512 | 3331.087802716296 | 3106.724409569968 |
| 225 | 3513.631724402148 | 3455.989687886776 | 3342.887663703478 | 3114.103693430824 |
| 226 | 3528.065802940997 | 3469.278526582705 | 3353.964420500084 | 3120.752512736096 |
| 227 | 3541.8154185539 | 3481.878742908683 | 3364.346322076875 | 3126.70024706716 |
| 228 | 3554.907660219622 | 3493.817644149496 | 3374.061125178855 | 3131.975738115214 |
| 229 | 3567.369091955182 | 3505.122003790013 | 3383.1360343575 | 3136.607230505484 |
| 230 | 3579.225701338927 | 3515.81801023141 | 3391.597651097503 | 3140.622321812966 |
| 231 | 3590.502856402031 | 3525.931223889967 | 3399.471931354446 | 3144.047921071538 |
| 232 | 3601.22527022094 | 3535.486542008432 | 3406.784150828181 | 3146.910215087767 |
| 233 | 3611.416972558195 | 3544.508170525054 | 3413.558877312272 | 3149.234641887304 |
| 234 | 3621.101287919594 | 3553.01960236611 | 3419.819949480997 | 3151.045870643984 |
| 235 | 3630.300819420638 | 3561.043601552977 | 3425.59046150106 | 3152.367787468418 |
| 236 | 3639.037437883631 | 3568.602192543363 | 3430.89275288409 | 3153.223486462738 |
| 237 | 3647.332275617531 | 3575.716654257272 | 3435.748403027369 | 3153.635265480484 |
| 238 | 3655.205724365032 | 3582.407518270781 | 3440.178229923094 | 3153.624626064284 |
| 239 | 3662.677436934572 | 3588.694570694145 | 3444.202292550145 | 3153.212277068664 |
| 240 | 3669.766332068488 | 3594.596857284337 | 3447.839896496375 | 3152.418141509908 |
| 241 | 3676.49060213178 | 3600.132691375604 | 3451.109602393107 | 3151.261366219425 |
| 242 | 3682.867723238678 | 3605.319664244383 | 3454.029236776506 | 3149.760333910776 |
| 243 | 3688.914467465896 | 3610.174657556806 | 3456.615905022721 | 3147.932677303218 |
| 244 | 3694.646916832018 | 3614.713857577641 | 3458.886006034452 | 3145.79529497608 |
| 245 | 3700.080478751674 | 3618.952770848862 | 3460.855248386154 | 3143.364368658295 |
| 246 | 3705.229902700834 | 3622.906241073739 | 3462.538667662979 | 3140.65538168581 |
| 247 | 3710.109297855637 | 3626.588466968586 | 3463.950644754964 | 3137.683138386398 |
| 248 | 3714.732151491659 | 3630.01302086881 | 3465.104924892595 | 3134.461784176455 |
| 249 | 3719.111347953358 | 3633.192867898789 | 3466.01463723294 | 3131.004826177712 |
| 250 | 3723.259188024568 | 3636.140385536368 | 3466.692314826845 | 3127.325154183478 |
| 251 | 3727.187408550527 | 3638.867383422327 | 3467.149914817451 | 3123.43506182398 |
| 252 | 3730.90720217992 | 3641.385123283292 | 3467.398838738397 | 3119.34626779881 |
| 253 | 3734.42923711189 | 3643.704338853052 | 3467.449952796697 | 3115.069937061284 |
| 254 | 3737.763676748088 | 3645.835255692348 | 3467.313608040462 | 3110.616701854829 |
| 255 | 3740.920199163471 | 3647.787610820956 | 3466.999660325389 | 3105.996682515575 |
| 256 | 3743.908016322036 | 3649.570672088287 | 3466.51749000647 | 3101.219507967851 |
| 257 | 3746.735892974838 | 3651.193257220006 | 3465.876021292613 | 3096.294335850734 |
| 258 | 3749.412165187773 | 3652.663752488227 | 3465.083741212043 | 3091.229872224042 |
| 259 | 3751.944758455616 | 3653.99013096197 | 3464.148718145406 | 3086.034390811326 |
| 260 | 3754.341205366927 | 3655.179970302584 | 3463.07861989166 | 3080.715751745609 |
| 261 | 3756.608662791586 | 3656.240470076092 | 3461.880731239003 | 3075.281419790904 |
| 262 | 3758.753928569142 | 3657.178468560815 | 3460.561971019542 | 3069.738482018983 |
| 263 | 3760.783457681772 | 3658.000459034214 | 3459.12890863204 | 3064.093664926566 |
| 264 | 3762.70337790059 | 3658.712605527878 | 3457.587780022006 | 3058.353350983066 |
| 265 | 3764.51950489841 | 3659.320758043965 | 3455.944503112808 | 3052.523594603431 |
| 266 | 3766.237356825824 | 3659.830467230088 | 3454.204692685181 | 3046.610137544335 |
| 267 | 3767.862168350728 | 3660.246998513001 | 3452.373674705875 | 3040.61842372538 |
| 268 | 3769.398904164253 | 3660.5753456942 | 3450.45650010888 | 3034.553613479591 |
| 269 | 3770.852271958453 | 3660.820244012948 | 3448.457958035176 | 3028.420597240092 |
| 270 | 3772.226734883161 | 3660.986182684368 | 3446.382588538925 | 3022.224008671701 |
| 271 | 3773.526523491107 | 3661.077416921787 | 3444.234694769709 | 3015.968237257968 |
| 272 | 3774.755647181836 | 3661.097979454114 | 3442.018354641992 | 3009.657440355551 |
| 273 | 3775.917905156131 | 3661.05169155006 | 3439.737432003672 | 3003.295554728958 |
| 274 | 3777.016896893557 | 3660.942173562 | 3437.395587317356 | 2996.886307579561 |
| 275 | 3778.056032166483 | 3660.77285500301 | 3434.996287867691 | 2990.433227083538 |
| 276 | 3779.03854060452 | 3660.54698417111 | 3432.542817509421 | 2983.939652453865 |
| 277 | 3779.96748082365 | 3660.267637335192 | 3430.038285970822 | 2977.408743541861 |
| 278 | 3780.845749134681 | 3659.93772749736 | 3427.485637727572 | 2970.843489994037 |
| 279 | 3781.676087845682 | 3659.560012746512 | 3424.887660462135 | 2964.246719980053 |
| 280 | 3782.461093173241 | 3659.137104218107 | 3422.246993123891 | 2957.621108507662 |
| 281 | 3783.203222777218 | 3658.671473674922 | 3419.566133605153 | 2950.96918534037 |
| 282 | 3783.904802933631 | 3658.165460723571 | 3416.847446047805 | 2944.293342533432 |
| 283 | 3784.568035360115 | 3657.621279681316 | 3414.093167795815 | 2937.595841603583 |
| 284 | 3785.19500370813 | 3657.04102610748 | 3411.305416007685 | 2930.878820347588 |
| 285 | 3785.787679735859 | 3656.426683013507 | 3408.486193943383 | 2924.14429932445 |
| 286 | 3786.347929175438 | 3655.780126765374 | 3405.637396939576 | 2917.394188015717 |
| 287 | 3786.877517307696 | 3655.103132691716 | 3402.760818086762 | 2910.630290677936 |
| 288 | 3787.378114257462 | 3654.3973804107 | 3399.858153621493 | 2903.854311901008 |
| 289 | 3787.851300021823 | 3653.664458888224 | 3396.931008046468 | 2897.067861885615 |
| 290 | 3788.298569243575 | 3652.9058712397 | 3393.980898990952 | 2890.27246145264 |
| 291 | 3788.721335741532 | 3652.123039287202 | 3391.00926182342 | 2883.46954679698 |
| 292 | 3789.120936809065 | 3651.31730788342 | 3388.017454028062 | 2876.66047399752 |
| 293 | 3789.498637291766 | 3650.489949013359 | 3385.006759356253 | 2869.846523295227 |
| 294 | 3789.855633454747 | 3649.642165684431 | 3381.978391763712 | 2863.02890314997 |
| 295 | 3790.193056649676 | 3648.775095615032 | 3378.933499143685 | 2856.208754087024 |
| 296 | 3790.51197679127 | 3647.889814731425 | 3375.873166865992 | 2849.3871523434 |
| 297 | 3790.813405652508 | 3646.98734048228 | 3372.798421131502 | 2842.56511332378 |
| 298 | 3791.09829998753 | 3646.06863497981 | 3369.710232151058 | 2835.743594875491 |
| 299 | 3791.367564490716 | 3645.134607976168 | 3366.60951715759 | 2828.923500391488 |
| 300 | 3791.622054600145 | 3644.18611968323 | 3363.497143259752 | 2822.105681749938 |
| 301 | 3791.862579153212 | 3643.223983443698 | 3360.373930145006 | 2815.290942098631 |
| 302 | 3792.08990290188 | 3642.248968260988 | 3357.24065263978 | 2808.480038492053 |
| 303 | 3792.304748894678 | 3641.261801195084 | 3354.098043133946 | 2801.673684388621 |
| 304 | 3792.50780073223 | 3640.263169631136 | 3350.946793876525 | 2794.8725520152 |
| 305 | 3792.699704702787 | 3639.253723427406 | 3347.787559149236 | 2788.077274605721 |
| 306 | 3792.881071803954 | 3638.2340769487 | 3344.620957324153 | 2781.288448520373 |
| 307 | 3793.05247965644 | 3637.2048109912 | 3341.447572811462 | 2774.506635251558 |
| 308 | 3793.21447431547 | 3636.166474604366 | 3338.267957903023 | 2767.73236332246 |
| 309 | 3793.36757198516 | 3635.119586815206 | 3335.08263451714 | 2760.966130083844 |
| 310 | 3793.51226064091 | 3634.06463826005 | 3331.892095849717 | 2754.208403414384 |
| 311 | 3793.649001564635 | 3633.002092728645 | 3328.696807936671 | 2747.459623329576 |
| 312 | 3793.778230797423 | 3631.932388625185 | 3325.497211132287 | 2740.720203504038 |
| 313 | 3793.900360513921 | 3630.855940350631 | 3322.293721507937 | 2733.990532711777 |
| 314 | 3794.015780322634 | 3629.773139610491 | 3319.086732175333 | 2727.27097618871 |
| 315 | 3794.124858496009 | 3628.684356652001 | 3315.876614538362 | 2720.561876921616 |
| 316 | 3794.227943134064 | 3627.589941434427 | 3312.663719477237 | 2713.863556867363 |
| 317 | 3794.325363265028 | 3626.490224736073 | 3309.448378468593 | 2707.176318106158 |
| 318 | 3794.417429886438 | 3625.385519201335 | 3306.230904644913 | 2700.500443932305 |
| 319 | 3794.504436949787 | 3624.276120331017 | 3303.011593796553 | 2693.836199885823 |
| 320 | 3794.58666229177 | 3623.162307418929 | 3299.790725319396 | 2687.18383472809 |
| 321 | 3794.664368514982 | 3622.04434443764 | 3296.568563111065 | 2680.543581364483 |
| 322 | 3794.73780382079 | 3620.922480876132 | 3293.345356418457 | 2673.91565771688 |
| 323 | 3794.807202796916 | 3619.796952531906 | 3290.121340639187 | 2667.300267548713 |
| 324 | 3794.872787162185 | 3618.66798226 | 3286.896738079452 | 2660.69760124511 |
| 325 | 3794.93476647075 | 3617.535780681245 | 3283.671758670628 | 2654.107836550561 |
| 326 | 3794.993338777948 | 3616.400546851958 | 3280.446600646849 | 2647.531139266383 |
| 327 | 3795.048691269893 | 3615.26246889712 | 3277.221451185658 | 2640.967663910165 |
| 328 | 3795.10100085874 | 3614.121724609067 | 3273.996487013729 | 2634.417554339234 |
| 329 | 3795.150434745463 | 3612.978482013486 | 3270.77187497955 | 2627.88094434011 |
| 330 | 3795.197150951956 | 3611.832899904566 | 3267.547772594857 | 2621.357958185752 |
| 331 | 3795.241298824061 | 3610.685128350905 | 3264.324328546495 | 2614.848711162392 |
| 332 | 3795.283019507134 | 3609.535309173796 | 3261.10168318034 | 2608.35331006756 |
| 333 | 3795.322446395622 | 3608.383576399383 | 3257.879968958758 | 2601.87185368088 |
| 334 | 3795.359705558073 | 3607.230056686098 | 3254.659310893086 | 2595.404433209128 |
| 335 | 3795.394916138904 | 3606.074869728718 | 3251.439826952444 | 2588.951132706937 |
| 336 | 3795.428190738201 | 3604.9181286404 | 3248.22162845019 | 2582.512029474466 |
| 337 | 3795.459635770732 | 3603.759940313619 | 3245.004820409238 | 2576.087194433311 |
| 338 | 3795.489351805322 | 3602.6004057616 | 3241.789501907384 | 2569.676692481818 |
| 339 | 3795.517433885644 | 3601.439620440948 | 3238.57576640372 | 2563.280582830935 |
| 340 | 3795.543971833443 | 3600.27767455648 | 3235.363702047167 | 2556.898919321658 |
| 341 | 3795.569050535165 | 3599.114653349626 | 3232.15339196814 | 2550.531750725075 |
| 342 | 3795.592750212864 | 3597.950637370826 | 3228.944914554193 | 2544.17912102596 |
| 343 | 3795.615146680284 | 3596.78570273708 | 3225.738343710591 | 2537.841069690816 |
| 344 | 3795.636311584901 | 3595.619921375375 | 3222.533749106593 | 2531.517631921205 |
| 345 | 3795.656312636685 | 3594.453361252758 | 3219.33119640822 | 2525.208838893187 |
| 346 | 3795.675213824341 | 3593.286086593807 | 3216.13074749828 | 2518.91471798361 |
| 347 | 3795.693075619676 | 3592.118158086174 | 3212.93246068432 | 2512.635292983985 |
| 348 | 3795.709955170762 | 3590.949633074841 | 3209.736390895172 | 2506.370584302611 |
| 349 | 3795.725906484508 | 3589.780565745726 | 3206.542589866709 | 2500.120609155599 |
| 350 | 3795.740980599211 | 3588.611007299288 | 3203.35110631743 | 2493.885381747414 |
| 351 | 3795.755225747635 | 3587.441006114407 | 3200.16198611439 | 2487.664913441472 |
| 352 | 3795.768687511163 | 3586.270607903532 | 3196.975272430037 | 2481.459212921394 |
| 353 | 3795.781408965447 | 3585.09985585913 | 3193.791005890437 | 2475.268286343375 |
| 354 | 3795.793430818112 | 3583.928790792149 | 3190.609224715357 | 2469.092137480181 |
| 355 | 3795.804791538863 | 3582.757451262903 | 3187.429964850674 | 2462.930767857232 |
| 356 | 3795.81552748247 | 3581.585873704731 | 3184.253260093487 | 2456.784176881197 |
| 357 | 3795.825673004992 | 3580.414092540908 | 3181.079142210392 | 2450.652361961515 |
| 358 | 3795.835260573616 | 3579.242140295095 | 3177.907641049238 | 2444.535318625234 |
| 359 | 3795.844320870477 | 3578.070047695762 | 3174.738784644753 | 2438.433040625533 |
| 360 | 3795.85288289076 | 3576.897843774837 | 3171.572599318371 | 2432.345520044252 |Blood IL-6 levels
(min)
Additional file 5, Figure E3
